# Discordance Between FIB-4 and BAST Fibrosis Risk Classifications in Obese Patients with MASLD: Results from the OBREDI-TR Study

**DOI:** 10.3390/diagnostics16040547

**Published:** 2026-02-12

**Authors:** Ozge Kama Basci, Alihan Oral, Ali Kirik, Hacer Sen, Ihsan Solmaz, Ulas Serkan Topaloglu, Ismail Demir, Ahmet Dundar, Emine Binnetoglu, Nalan Okuroglu, Ahmet Aydin, Zeynep Irmak Kaya, Hamit Yildiz, Aycan Acet, Gokhan Tazegul, Osman Ozudogru, Kubilay Issever, Selcuk Yaylacı, Ugur Bayram Korkmaz, Nur Duzen Oflas, Celalettin Küçük, Kamil Konur, Teslime Ayaz, Aysun Isiklar, Esref Arac, Hilmi Erdem Sumbul, Hüseyin Ali Öztürk, Ali Burak Govez, Yusuf Usame Durmus, Atilla Onmez, Sibel Ocak Serin, Nizameddin Koca, Nazif Yalcin, Aysegul Ertinmaz, Alper Tuna Guven, Mehmet Kok, Yasin Sahinturk, Seyit Uyar, Hasan Sözel

**Affiliations:** 1 Department of Internal Medicine, Faculty of Medicine, Balikesir University, Altieylül, Balikesir 10145, Türkiye; ozgee.kama@gmail.com (O.K.B.); alikirik87@hotmail.com (A.K.); hcrgrsy@hotmail.com (H.S.); 2 Department of Internal Medicine, Faculty of Medicine, Biruni University, Halkalı Street No. 99, Istanbul 34295, Türkiye; dr.alihanoral@gmail.com; 3 Department of Internal Medicine, Diyarbakir Gazi Yasargil Education Research Hospital, Diyarbakir 21070, Türkiye; ihsan2157@gmail.com; 4 Department of Internal Medicine, Kayseri City Hospital, Kayseri 38080, Türkiye; ustop38@gmail.com; 5 Department of Internal Medicine, Bozyaka Education Research Hospital, Izmir 35170, Türkiye; drismaildemir22@gmail.com; 6 Department of Internal Medicine, Mardin Savur Prof. Dr. Aziz Sancar State Hospital, Savur 47860, Türkiye; drahmetdundar@hotmail.com; 7 Department of Internal Medicine, Corlu Vatan Hospital, Corlu 59860, Türkiye; edemirbas1@yahoo.com; 8 Department of Internal Medicine, Fatih Sultan Mehmet Education Research Hospital, Istanbul 34752, Türkiye; nokuroglu@yahoo.com; 9 Department of Internal Medicine, Faculty of Medicine, Medipol University, Bagcilar, Istanbul 34214, Türkiye; uzm.dr.ahmetaydin@gmail.com; 10 Department of Internal Medicine, Health Sciences University Eskisehir Health Application and Research Center, Eskisehir City Hospital, Eskisehir 26080, Türkiye; dr.zeynepirmak@gmail.com; 11 Department of Internal Medicine, Faculty of Medicine, Gaziantep University, Sehitkamil 27600, Türkiye; drhyildiz@haotmail.com; 12 Department of Internal Medicine, Faculty of Medicine, Kutahya Health Sciences University, Kutahya 43020, Türkiye; aycanacet80@icloud.com; 13 Department of Internal Medicine, Faculty of Medicine, Marmara University, Istanbul 34854, Türkiye; drgtazegul@gamil.com; 14 Department of Internal Medicine, Faculty of Medicine, Erzincan Binali Yildirim University, Erzincan 24100, Türkiye; osmanozudogru2@gmail.com; 15 Department of Internal Medicine, Giresun University Education Research Hospital, Giresun 28100, Türkiye; kubilayissever@gmail.com; 16 Department of Internal Medicine, Faculty of Medicine, Sakarya University, Sakarya 54100, Türkiye; selcukyaylaci@sakarya.edu.tr; 17 Department of Internal Medicine, Izmir Katip Celebi Education Research Hospital, Izmir 35360, Türkiye; ugurbk07@gmail.com; 18 Department of Internal Medicine, Faculty of Medicine, Van Yuzuncu Yil University, Van 54100, Türkiye; dr.nurdzn@hotmail.com; 19 Bahçelievler Memorial Hospital, Istanbul 34180, Türkiye; celalettinkucuk@yahoo.com; 20 Department of Internal Medicine, Faculty of Medicine, Recep Tayyip Erdogan University, Rize 53020, Türkiye; kamil.konur@erdogan.edu.tr; 21 Department of Internal Medicine, Bakircay University Cigli Education Research Hospital, Izmir 36610, Türkiye; drteslimeayaz@gmail.com; 22 Department of Internal Medicine, Acibadem Atasehir Hospital, Atasehir, Istanbul 34642, Türkiye; aysunisiklar@gmail.com; 23 Department of Internal Medicine, Faculty of Medicine, Dicle University, Diyarbakir 21010, Türkiye; esrefarac@gmail.com; 24 Department of Internal Medicine, Health Sciences University Adana Health Application and Research Center, Adana City Hospital, Adana 01230, Türkiye; esumbul@cu.edu.tr (H.E.S.); drozturkhuseyinali@gmail.com (H.A.Ö.); abgovez@gmail.com (A.B.G.); drmusyusuf80@gmail.com (Y.U.D.); 25 Department of Internal Medicine, Faculty of Medicine, Duzce University, Duzce 81000, Türkiye; attilaonmez@gmail.com; 26 Department of Internal Medicine, Umraniye Education Research Hospital, Umraniye, Istanbul 34764, Türkiye; rdsibelocak@gmail.com; 27 Department of Internal Medicine, Health Sciences University Bursa Health Application and Research Center, Bursa City Hospital, Bursa 16250, Türkiye; nkoca@yahoo.com (N.K.); nazifyalcin16@gmail.com (N.Y.); aertinmaz@yahoo.com (A.E.); 28 Department of Internal Medicine, Faculty of Medicine, Baskent University, Ankara 06790, Türkiye; alper.tuna.guven@gmail.com; 29 Department of Internal Medicine, Antalya Education Research Hospital, Antalya 07080, Türkiye; dr.mehmetkok@hotmail.com (M.K.); drsahinturk@yahoo.com (Y.S.); seyituyar79@hotmail.com (S.U.); 30 Department of Internal Medicine, Faculty of Medicine, Akdeniz University, Antalya 07100, Türkiye

**Keywords:** obesity, MASLD, FIB-4 score, BAST score, liver fibrosis

## Abstract

**Background/Objectives**: Non-invasive fibrosis scores are widely used for risk stratification in metabolic dysfunction-associated steatotic liver disease (MASLD); however, their performance in obese individuals remains controversial. The Fibrosis-4 (FIB-4) index is commonly recommended as a first-line tool, yet it may underestimate fibrosis risk in severe obesity. The BAST score, which incorporates metabolic and anthropometric parameters, has been proposed as an alternative. This study aimed to characterize both the degree and direction of discordance between FIB-4 and BAST in obese patients with MASLD. **Methods**: This predefined secondary analysis included 2950 adults with obesity (BMI ≥ 30 kg/m^2^) and MASLD from the multicenter OBREDI-TR cohort. Fibrosis risk categories were assigned using standard cut-offs for FIB-4 and BAST, and agreement was assessed using weighted Cohen’s kappa. Associations among discordance patterns, obesity class, and the visceral adiposity index (VAI) were evaluated using chi-square tests and general linear models. **Results**: Overall agreement between FIB-4 and BAST was very poor (κ = 0.041, *p* < 0.001). Discordance was observed in 22.3% of patients and increased markedly with obesity severity. In class III obesity, discordance was predominantly driven by low-risk classification according to FIB-4 despite high-risk classification according to BAST. Patients with this discordant pattern exhibited significantly higher VAI values than concordant cases (*p* < 0.001), independently of the study center. **Conclusions**: In obese patients with MASLD, particularly those with morbid obesity, FIB-4 frequently classifies patients as low risk, while BAST identifies elevated fibrosis risk. This systematic discordance suggests that FIB-4 may underestimate fibrosis burden in the context of severe obesity and visceral adiposity, supporting the need for a phenotype-oriented, multimodal approach to fibrosis risk assessment.

## 1. Introduction

Metabolic dysfunction-associated steatotic liver disease (MASLD) has emerged as a major public health challenge owing to its rapidly increasing global prevalence. Current epidemiological evidence indicates that nearly one-third of the world’s population exhibits liver alterations related to MASLD, closely paralleling the global epidemics of obesity and insulin resistance [1,2].

The 2025 Global Consensus on metabolic dysfunction-associated steatotic liver disease and steatohepatitis (MASLD/MASH) represented a paradigm shift in diagnostic strategy by stating that ultrasonography is no longer a mandatory criterion for MASLD diagnosis and that non-invasive tests (NITs) should instead be prioritized to assess metabolic risk profile, biochemical abnormalities, and fibrosis severity [3]. These NITs include serum-based fibrosis scores such as the Fibrosis-4 (FIB-4) index, the NAFLD Fibrosis Score (NFS), and the Enhanced Liver Fibrosis (ELF) test; elastography-based techniques, including vibration-controlled transient elastography (VCTE) and magnetic resonance elastography (MRE); and composite algorithms that integrate biochemical and imaging parameters, such as the FibroScan-AST (FAST) score, Agile 3+, and the combination of magnetic resonance elastography and FIB-4 (MEFIB) [4,5].

Among these, FIB-4 is an inexpensive and widely accessible first-line tool calculated using age, AST, ALT, and platelet count, and it demonstrates a high negative predictive value for excluding advanced fibrosis (F3–F4) [6]. Large cohort studies have reported AUROC values of approximately 0.78–0.85 for advanced fibrosis detection [7]. However, its ability to identify early fibrosis stages (F0–F2) and fibrotic MASH remains limited, and the broad indeterminate “gray zone” frequently necessitates secondary testing [8].

Importantly, the diagnostic performance of NITs deteriorates in metabolically high-risk populations. Both FIB-4 and the NFS exhibit substantial rates of false negativity and false positivity, with false-negative results occurring more frequently in individuals with metabolic risk factors [9]. In a large NHANES-based analysis, approximately 10% of individuals at risk of MASLD who were categorized as “low risk” based on FIB-4 demonstrated significant liver stiffness (≥8 kPa) on VCTE. This misclassified low-risk group had higher BMI, waist circumference, and diabetes prevalence [10]. Similarly, a national multicenter MASLD study showed that the prevalence of elevated FIB-4 scores declined from 28.1% to 8.7% with the increase in BMI, suggesting systematic underestimation of fibrosis risk in obese individuals [11]. In that study, higher platelet counts observed in obese and smoking young individuals further contributed to lower FIB-4 values.

In morbidly obese NAFLD patients, the discriminative ability of FIB-4, the NFS, and the AST-to-platelet ratio index (APRI) using conventional cut-off values is poor (AUROC < 0.62), limiting their utility primarily to the exclusion of advanced fibrosis [12]. BMI-stratified analyses in MAFLD populations have similarly demonstrated that FIB-4 and the NFS fail to accurately identify advanced fibrosis in underweight and morbidly obese individuals, showing acceptable performance only in their exclusion in overweight or non-morbidly obese patients [13,14]. Notably, in severely obese bariatric surgery candidates, the AUROC of FIB-4 in advanced fibrosis detection using standard thresholds is as low as 0.57, and failure to apply revised lower cut-offs results in a substantial proportion of advanced fibrosis cases being missed [15].

Age dependency, limited sensitivity in MASLD patients with normal transaminase levels, susceptibility to false-negative results in conditions associated with elevated platelet counts, and inability to capture key metabolic features such as BMI, insulin resistance, and hypertriglyceridemia are recognized as the principal limitations of FIB-4 [16,17,18].

In this context, the development of fibrosis risk scores incorporating metabolic determinants has gained increasing clinical relevance. The BAST score (Body mass index, Age, AST, and Triglycerides) is a novel non-invasive index that integrates four variables closely aligned with MASLD pathophysiology and has been proposed as a more accurate tool for fibrosis risk assessment, particularly in obese individuals [19]. In the study by Helal et al., BAST demonstrated markedly superior discriminative performance compared with FIB-4 (AUROC 0.90 vs. 0.61) and significantly reduced false-negative rates in advanced fibrosis detection. By directly incorporating obesity-related parameters, BAST offers a more reliable risk stratification in metabolically high-risk populations and appears more robust to confounding by age and platelet count [19].

Accordingly, this study aims to test the hypothesis that the BAST score may serve as an alternative or complementary tool to FIB-4 for fibrosis risk assessment in obese individuals.

## 2. Materials and Methods

### 2.1. Study Design and Data Source

The present study was conducted as a predefined secondary analysis of the Obesity-Related Disorders in Türkiye (OBREDI-TR) study database [20]. OBREDI-TR is a large-scale, multicenter, retrospective, cross-sectional study performed across multiple tertiary care centers in Türkiye, designed to evaluate obesity-related metabolic and organ-specific complications under real-world clinical conditions.

This secondary analysis specifically focused on patients diagnosed with metabolic dysfunction-associated steatotic liver disease (MASLD) within the OBREDI-TR cohort. This study was conducted in accordance with the principles of the Declaration of Helsinki and was approved by the Ethics Committee of Biruni University (approval number 2024/84; date: 19 November 2024).

### 2.2. Definition of MASLD

MASLD was diagnosed according to the 2024 EASL–EASD–EASO consensus criteria, defined by the presence of hepatic steatosis detected by abdominal ultrasonography together with at least one cardiometabolic risk factor [21]. Metabolic dysfunction was defined according to the presence of obesity-related metabolic abnormalities, including dysglycemia, dyslipidemia, hypertension, or insulin resistance, as documented in the clinical records. Significant alcohol consumption was defined as an average daily intake exceeding 20 g/day in women and 30 g/day in men, and individuals meeting this criterion were excluded to ensure a metabolically driven disease phenotype. Additional exclusion criteria included chronic viral hepatitis, autoimmune liver disease, drug-induced liver injury, malignancy, pregnancy, and other secondary causes of hepatic steatosis.

### 2.3. Study Population

Adult patients aged ≥18 years with obesity, defined as a body mass index (BMI) ≥ 30 kg/m^2^, who fulfilled the diagnostic criteria for MASLD were eligible for inclusion. As all participants were individuals with obesity, the presence of at least one cardiometabolic risk factor required by the 2024 MASLD definition was inherently fulfilled.

Hepatic steatosis was assessed using abdominal ultrasonography performed as part of routine clinical evaluation. Individuals with known chronic liver diseases, including autoimmune hepatitis, established cirrhosis, chronic viral hepatitis, cholestatic liver diseases, and hereditary liver disorders, were not included in the cohort. Individuals with significant alcohol consumption were excluded based on standardized clinical history.

Patients with missing laboratory data required for the calculation of non-invasive fibrosis scores were excluded. Further exclusion criteria included pregnancy, acute or chronic inflammatory diseases, active malignancy, history of bariatric surgery, and chronic liver disease due to secondary etiologies. After the application of all inclusion and exclusion criteria, we included 2950 patients in the final analysis. Participants were stratified according to obesity severity based on BMI categories as follows:

Obesity class I: BMI of 30.0–34.9 kg/m^2^.

Obesity class II: BMI of 35.0–39.9 kg/m^2^.

Obesity class III: BMI ≥ 40.0 kg/m^2^.

Clinical and Laboratory Parameters

Anthropometric measurements, including BMI and waist circumference (WC), were recorded using standardized procedures. The presence of obesity-related comorbidities, including type 2 diabetes mellitus (T2DM), hypertension, dyslipidemia, metabolic syndrome, and obesity, was documented based on clinical evaluation and medical records.

Laboratory assessments included complete blood count parameters, aspartate aminotransferase (AST), alanine aminotransferase (ALT), total cholesterol, triglycerides (TG), and high-density-lipoprotein cholesterol (HDL-C).

### 2.4. Non-Invasive Fibrosis Scores

The FIB-4 index was calculated using the following formula:

FIB-4 index = Age (years) × AST (U/L)/[Platelet count (10^9^/L) × √ALT (U/L)].

Based on established cut-off values, patients were categorized into three fibrosis risk groups [22]:

Low risk: FIB-4 < 1.30.

Intermediate risk: FIB-4 of 1.30–2.67.

High risk: FIB-4 > 2.67.

The new BAST score was calculated using the following equation:

BAST score = 0.086 × waist circumference (cm) + 0.08 × body mass index (kg/m^2^) + 0.025 × AST (IU/L) − 14.607

Patients were stratified into fibrosis risk categories according to predefined BAST cut-off values [19]:

Low risk: BAST < 1.48.

Intermediate risk: BAST of 1.48–2.59.

High risk: BAST > 2.59.

Visceral Adiposity Index

Visceral adiposity was assessed using the visceral adiposity index (VAI), a sex-specific surrogate marker of visceral fat distribution and adipose tissue dysfunction [23].

For women, the VAI was calculated as

VAI = [WC/(36.58 + 1.89 × BMI)] × (TG/0.81) × (1.52/HDL-C).

For men, the VAI was calculated as

(1) VAI = [WC/(39.68 + 1.88 × BMI)] × (TG/1.03) × (1.31/HDL-C).

Waist circumference was expressed in centimeters, and TG and HDL-C values were expressed in mmol/L. Based on the previous literature, a VAI value of approximately 1.0 was considered indicative of visceral adipose tissue dysfunction. For analytical purposes, the VAI was treated as a continuous variable, with higher values reflecting increasing degrees of visceral adiposity and metabolic risk.

### 2.5. Statistical Analysis

Continuous variables were expressed as means ± standard deviations, and categorical variables were presented as counts and percentages. The normality of continuous variables was assessed using the Kolmogorov–Smirnov test. Between-group comparisons were performed using independent-sample *t*-tests for continuous variables and chi-square tests for categorical variables, as appropriate.

Agreement between fibrosis risk classifications derived from the FIB-4 index and the BAST score was evaluated using Cohen’s weighted kappa (κ) statistics. Based on the degree of agreement, patients were classified as concordant when both scoring systems assigned the same fibrosis risk category and discordant when the classifications differed.

Associations between discordance status and obesity severity were assessed using chi-square tests, with effect size being quantified with Cramer’s V. Differences in the VAI between concordant and discordant groups were initially evaluated using independent-sample *t*-tests.

To account for the multicenter structure of the OBREDI-TR dataset, general linear models (GLMs) were estimated using a complex sampling design, with patients clustered within centers. This approach accounts for intracenter correlation by applying design-based variance estimation with robust standard errors, ensuring valid statistical inference in the presence of clustered observations. No random effects were specified. In these models, the visceral adiposity index (VAI) was included as the dependent variable, and the fibrosis score discordance status was included as the fixed factor. Model estimates were reported with corresponding test statistics.

All statistical analyses were performed using IBM SPSS Statistics, version 30 (IBM Corp., Armonk, NY, USA). A two-sided *p*-value < 0.05 was considered statistically significant.

## 3. Results

### 3.1. Baseline Characteristics

A total of 2950 patients with obesity and MASLD were included in the final analysis, comprising 2108 women (71.5%) and 842 men (28.5%). The overall mean age of the study population was 45.15 ± 13.58 years. The mean body mass index (BMI) was 37.34 ± 6.12 kg/m^2^, corresponding to class II obesity. Detailed demographic, anthropometric, and laboratory characteristics of the study population, stratified by sex, are presented in Table 1.

### 3.2. Agreement Between FIB-4 and BAST Classifications

The agreement between fibrosis risk categories derived from the FIB-4 index and the BAST score was assessed using Cohen’s weighted kappa (κ) statistics. The analysis demonstrated very poor agreement between the two scoring systems (κ = 0.041, *p* < 0.001), indicating substantial discordance in fibrosis risk classification (Table 2).

Among patients classified as concordant, agreement between FIB-4 and BAST was predominantly observed in the low fibrosis risk category. Most concordant cases were jointly classified as low risk by both scoring systems, whereas concordance within the intermediate and high fibrosis risk categories was relatively infrequent. Accordingly, overall agreement between FIB-4 and BAST was largely attributable to shared low-risk classification, with higher fibrosis risk categories accounting for a smaller proportion of concordant cases.

Based on these findings, a discordance variable was generated. Of the 2950 participants, 2292 (77.7%) were classified as concordant, while 658 patients (22.3%) were classified as discordant with respect to FIB-4 and BAST fibrosis risk categories.

### 3.3. Association Between Obesity Severity and Discordance

**Figure 1:**
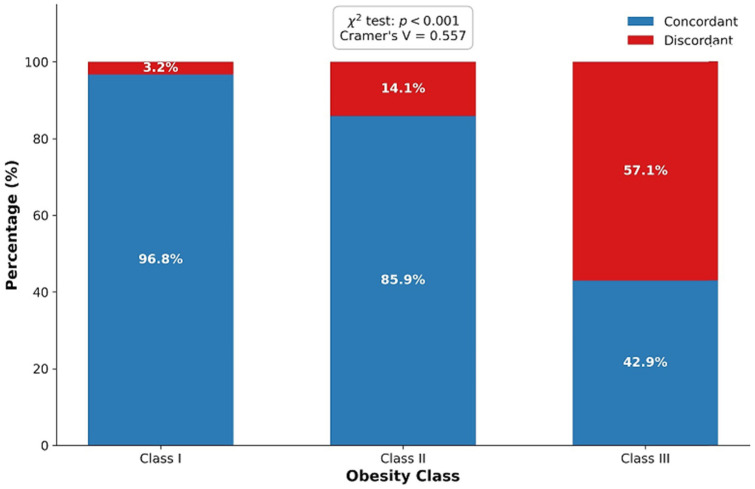
Association between obesity class and FIB-4–BAST discordance.

A strong and statistically significant association was observed between fibrosis score discordance and obesity severity. Discordance rates increased markedly across obesity classes (χ^2^(2, N = 2950) = 914.38, *p* < 0.001), with a large effect size (Cramer’s V = 0.557). As shown in Figure 1, 76.7% of discordant cases were observed in patients with obesity class III, compared with only 16.6% among concordant cases.

### 3.4. Visceral Adiposity and Discordance

Visceral adiposity index (VAI) values differed significantly between concordant and discordant groups. Patients in the discordant group exhibited substantially higher VAI levels compared with those in the concordant group (means ± SDs: 1639.43 ± 1250.13 vs. 1162.81 ± 1048.25, respectively). This difference was statistically significant (t(912.01) = 8.80, *p* < 0.001) and corresponded to a small-to-moderate effect size (Cohen’s d = 0.44).

To account for the multicenter structure of the dataset, a general linear model (GLM) was applied with patients nested within centers. In this model, the VAI was included as the dependent variable and discordance status as the fixed factor. The GLM confirmed a significant association between discordance and higher VAI values (Wald F(1, 27) = 96.723, *p* < 0.001), explaining 3.2% of the variance in the VAI. Discordant classification was associated with an estimated mean increase of 476.63 units in the VAI (B = −476.63, t(27) = −9.84, *p* < 0.001). These findings corroborated the results of the unadjusted analyses.

## 4. Discussion

In this study, we observed very poor agreement between two commonly used non-invasive fibrosis scores, FIB-4 and BAST, in patients with obesity and MASLD. The marked discordance demonstrated by Cohen’s weighted kappa analysis suggests that these two scores may prioritize different patient subgroups when classifying fibrosis risk. Consistent with the existing literature, our findings indicate that FIB-4 and BAST are not interchangeable tools in the obese MASLD population; rather, they appear to reflect distinct clinical and metabolic phenotypes [19].

Although limited agreement among non-invasive fibrosis tests has been previously reported in the literature, most prior studies were conducted in heterogeneous populations or were primarily designed to compare these tools against elastography-based methods. Data specifically addressing discordance between FIB-4 and newly developed fibrosis scores in large, obesity-focused cohorts—particularly among individuals with MASLD—remain scarce [24]. In this context, findings derived from the OBREDI-TR cohort suggest that fibrosis risk stratification in obese patients with MASLD may require a more nuanced and phenotype-oriented approach.

In our study, discordance in fibrosis risk classification was found to be particularly pronounced among patients with class III obesity. The observation that the majority of discordant cases occurred in individuals with morbid obesity suggests that the severity of obesity may substantially influence the results of non-invasive fibrosis scores. These findings indicate that scores such as FIB-4 and BAST do not exhibit homogeneous performance across the obese MASLD population and that divergence between scores becomes more evident at higher levels of obesity severity [12].

Similarly, comparative studies using elastography-based methods have demonstrated that in individuals with morbid obesity, reliance on a single non-invasive test may be insufficient for accurate fibrosis assessment. Several studies have reported that agreement between different fibrosis scoring systems decreases as obesity severity increases, highlighting the complexity of fibrosis risk stratification in this population [25,26]. These observations underscore the need for a more cautious interpretation of non-invasive fibrosis scores in advanced obesity and provide a relevant framework for understanding the pronounced discordance observed in our cohort.

Consistent with the existing literature, studies validated against elastography or liver biopsy have further shown that the performance of non-invasive fibrosis tests varies according to obesity stage. In patients with severe obesity, platelet-based scores such as FIB-4 tend to classify a greater proportion of individuals into lower fibrosis risk categories, whereas scores incorporating anthropometric parameters and measures of adiposity appear to identify a different subset of patients with potentially higher fibrosis risk [27]. This phenomenon has been attributed, at least in part, to the relative preservation of platelet counts and the limited sensitivity of aminotransferase levels for reflecting fibrosis severity in the context of advanced obesity [28].

Beyond obesity severity, visceral adiposity has been increasingly recognized as a key determinant of hepatic inflammation and fibrogenesis. Previous studies have demonstrated a close association between visceral fat accumulation and liver fibrosis severity, independent of overall body mass index, with visceral adiposity acting as a major driver of insulin resistance, systemic inflammation, and profibrotic signaling within the liver [29]. In this regard, the visceral adiposity index (VAI) has been proposed as a surrogate marker of dysfunctional visceral fat and has been shown to correlate with fibrosis severity in patients with MASLD and related metabolic liver diseases [30].

In the present study, patients exhibiting discordant fibrosis risk classification—characterized by higher BAST scores but lower FIB-4 values—also demonstrated significantly higher VAI levels, particularly among those with morbid obesity. Although our study does not allow conclusions regarding the diagnostic superiority of one fibrosis score over another to be drawn, this finding is noteworthy. It suggests that discordance between BAST and FIB-4 may, at least in part, reflect differences in the extent of visceral adiposity and its metabolic consequences. In this context, elevated VAI values in patients with high BAST but low FIB-4 scores may indicate a subgroup of obese individuals in whom visceral adipose dysfunction and fibrosis-related metabolic burden are more pronounced, despite relatively low platelet-based fibrosis estimates.

This study has several limitations that should be acknowledged. First, the absence of an external reference standard, such as vibration-controlled transient elastography or liver biopsy, precludes conclusions regarding the diagnostic superiority or true accuracy of FIB-4 or BAST for fibrosis staging. Accordingly, our findings should be interpreted as reflecting discordance in fibrosis risk stratification rather than definitive misclassification. Second, we applied guideline-recommended cut-off values for FIB-4, which are not specifically tailored to obese or morbidly obese populations. Future studies are warranted to explore obesity-adjusted cut-offs and to validate these thresholds against invasive or imaging-based fibrosis assessments.

In addition, both BAST and the visceral adiposity index (VAI) incorporate overlapping anthropometric and metabolic components, including body mass index and waist circumference. Therefore, it remains uncertain whether elevated BAST scores in morbidly obese individuals with low FIB-4 values primarily reflect increased hepatic fibrosis risk or instead capture heightened metabolic and visceral adiposity burden. Moreover, vascular imaging markers of subclinical atherosclerosis, such as carotid intima–media thickness or coronary atherosclerosis measures, were not available in the present cohort. This represents a limitation, as prior studies have demonstrated close associations between liver fibrosis severity in MASLD/NAFLD and carotid or coronary atherosclerotic burden. The lack of such data limited our ability to assess whether discordant fibrosis risk classifications were accompanied by parallel vascular structural changes. Finally, spleen-related parameters were not assessed. Although emerging evidence suggests an interaction between liver fibrosis severity and spleen-related changes in chronic liver disease, the absence of splenic measurements precluded exploration of this relationship in the current study. Prospective studies integrating elastography or histological endpoints, together with vascular imaging and organ-level assessments, will be essential to clarifying the clinical and mechanistic implications of discordant fibrosis risk classifications in obese patients with MASLD.

## 5. Conclusions

In this large, multicenter cohort of obese patients with MASLD, we demonstrate substantial and clinically meaningful discordance between FIB-4 and BAST fibrosis risk classifications. This discordance is not random but is predominantly characterized by low-risk categorization according to FIB-4 alongside high-risk categorization based on BAST, particularly in individuals with morbid obesity. The strong association between this discordant pattern and increased visceral adiposity underscores the influence of obesity-related metabolic phenotypes on non-invasive fibrosis assessment. Our findings suggest that platelet- and age-based scores such as FIB-4 may underestimate fibrosis risk in the setting of severe obesity, while scores incorporating anthropometric and metabolic parameters identify a distinct high-risk subgroup. These results emphasize the need for a phenotype-oriented approach to fibrosis risk stratification in obese MASLD patients and support the integration of complementary non-invasive tools rather than relying on a single score, especially in advanced obesity.

## Figures and Tables

**Table 1:** Baseline clinical and laboratory characteristics of the study population.

Variable	Females (n = 2108)	Males (n = 842)	Total (N = 2950)
Age, years	45.14 ± 13.64	45.16 ± 13.44	45.15 ± 13.58
Weight, kg	96.85 ± 16.62	109.68 ± 19.20	100.51 ± 18.33
Height, cm	159.92 ± 6.62	174.09 ± 7.59	163.97 ± 9.42
BMI, kg/m^2^	37.80 ± 6.20	36.19 ± 5.76	37.34 ± 6.12
Waist circumference, cm	110.93 ± 14.07	115.07 ± 13.78	112.11 ± 14.11
Systolic BP, mmHg	134.24 ± 15.43	136.64 ± 15.18	134.93 ± 15.39
Diastolic BP, mmHg	80.83 ± 8.22	81.64 ± 8.48	81.06 ± 8.30
Fasting glucose, mg/dL	118.10 ± 47.67	117.56 ± 45.85	117.95 ± 47.15
HbA1c, %	6.44 ± 1.52	6.48 ± 1.47	6.45 ± 1.51
Platelets, ×10^9^/L	279.14 ± 72.58	281.33 ± 76.46	279.76 ± 73.70
ALT, U/L	29.87 ± 22.61	31.94 ± 27.26	30.47 ± 24.04
AST, U/L	24.80 ± 18.84	24.88 ± 14.21	24.82 ± 17.64
Triglycerides, mg/dL	173.16 ± 104.31	171.50 ± 100.61	172.69 ± 103.25
HDL-C, mg/dL	48.33 ± 13.60	47.81 ± 12.43	48.18 ± 13.28

Abbreviations: ALT, alanine aminotransferase; AST, aspartate aminotransferase; BMI, body mass index; BP, blood pressure; HbA1c, glycated hemoglobin; HDL-C, high-density-lipoprotein cholesterol.

**Table 2:** Agreement between FIB-4 and BAST fibrosis risk categories.

FIB-4\BAST	Low	Intermediate	High
Low	1650	364	658
Intermediate	118	41	79
High	15	5	20
Total	1783	410	757

## Data Availability

The datasets used and analyzed during the current study are available from the corresponding author upon reasonable request.
